# Horizontal gene transfer and silver nanoparticles production in a new *Marinomonas* strain isolated from the Antarctic psychrophilic ciliate *Euplotes focardii*

**DOI:** 10.1038/s41598-020-66878-x

**Published:** 2020-06-23

**Authors:** Maria Sindhura John, Joseph Amruthraj Nagoth, Kesava Priyan Ramasamy, Patrizia Ballarini, Matteo Mozzicafreddo, Alessio Mancini, Andrea Telatin, Pietro Liò, Gabriele Giuli, Antonino Natalello, Cristina Miceli, Sandra Pucciarelli

**Affiliations:** 1 School of Bioscience and Veterinary Medicine, University of Camerino, Via Gentile III da Varano, 1, 62032 Camerino, Italy; 2 Quadram Institute Bioscience, Gut Microbes and Health Institute Strategic Program, Norwich Research Park, Norwich, UK; 3 Computer Laboratory, University of Cambridge, 15 JJ Thomson Avenue, Cambridge, UK; 4 School of Science and Technology, University of Camerino, Via Gentile III da Varano, 1, 62032 Camerino, Italy; 5 Department of Biotechnology and Biosciences, University of Milano-Bicocca, Piazza della Scienza, 2, 20126 Milano, Italy

**Keywords:** Environmental biotechnology, Ecological genetics

## Abstract

We isolated a novel bacterial strain from a prokaryotic consortium associated to the psychrophilic marine ciliate *Euplotes focardii*, endemic of the Antarctic coastal seawater. The 16S rDNA sequencing and the phylogenetic analysis revealed the close evolutionary relationship to the Antarctic marine bacterium *Marinomonas* sp. BSw10506 and the sub antarctic *Marinomonas polaris*. We named this new strain *Marinomonas* sp. ef1. The optimal growth temperature in LB medium was 22 °C. Whole genome sequencing and analysis showed a reduced gene loss limited to regions encoding for transposases. Additionally, five genomic islands, e.g. DNA fragments that facilitate horizontal gene transfer phenomena, were identified. Two open reading frames predicted from the genomic islands coded for enzymes belonging to the Nitro-FMN-reductase superfamily. One of these, the putative NAD(P)H nitroreductase YfkO, has been reported to be involved in the bioreduction of silver (Ag) ions and the production of silver nanoparticles (AgNPs). After the *Marinomonas* sp. ef1 biomass incubation with 1 mM of AgNO_3_ at 22 °C, we obtained AgNPs within 24 h. The AgNPs were relatively small in size (50 nm) and had a strong antimicrobial activity against twelve common nosocomial pathogenic microorganisms including *Staphylococcus aureus* and two *Candida* strains. To our knowledge, this is the first report of AgNPs biosynthesis by a *Marinomonas* strain. This biosynthesis may play a dual role in detoxification from silver nitrate and protection from pathogens for the bacterium and potentially for the associated ciliate. Biosynthetic AgNPs also represent a promising alternative to conventional antibiotics against common pathogens.

## Introduction

Antarctic coastal seawater houses a large number of microbial ecosystems. A broad portion of the eukaryotic microbes that inhabit the shallow water sediments of the Antarctic coasts is represented by ciliated protozoa (Ciliophora, Alveolata). Ciliates are ubiquitous heterotrophic unicellular eukaryotes that play a fundamental role in the “microbial loop^1^” in several ecological niches of all environments. Often considered as a natural microcosm, they offer habitats for bacteria association in different compartments, such as cytoplasm, nuclei and perinuclear spaces^2,3^, and cortical surface^4^. Ciliate features, the large size and the feeding habit based on phagocytosis, appear to be favorable traits for bacteria symbiosis relationships. These traits may provide a selective advantage in challenging and harsh ecosystems^5,6^. *Euplotes focardii* is a free-swimming endemic ciliate of Terra Nova Bay oligotrophic coastal sediments (Antarctica) (temperature, −1.8 °C; salinity, 35‰; pH, 8.1–8.2)^7^. In laboratory conditions, the optimal growth temperature of *E. focardii* is about 4–5 °C^8,9^. Therefore, it is classified as an obligate stenothermal psychrophilic microorganism. *E. focardii* psychrophilic phenotype also includes peculiar microtubules dynamics^10–13^ and enzyme biochemical properties^14–17^. The bacterial consortium associated with this organism has been characterized^18,19^. Here we describe a new *Marinomonas* strain isolated from the consortium and we report its genome characteristics. We identified genomic islands (GEIs), e.g. discrete mobile DNA segments involved in the propagation of virulence and antibiotic resistance genes, as well as catabolic genes leading to formation of new metabolic pathways^20^. GEIs propagation to new hosts occurs by transformation, conjugation or transduction. GEIs facilitate horizontal (or lateral) gene transfer phenomena, known to be implicated in environmental adaptation^21^.

The genus *Marinomonas* comprises gram negative aerobic bacteria previously classified in two different species: *Alteromonas vaga* and *Alteromonas communis*^22^. It is distributed in various marine environments and symbiotic species have been already identified^23^. Cold adapted species are *Marinomonas polaris* and *Marinomonas ushuaiensis*^24^. The new *Marinomonas* strain here described, named *Marinomonas* sp. ef1, is the first to be isolated from an Antarctic consortium^25^. *Marinomonas* sp. ef1 is able to produce silver nanoparticles (AgNPs) from silver nitrate at relatively low temperatures. NPs (Ø 1 to 100 nm) are used in different fields as healthcare, space industries, cosmetics, chemical industries, optoelectronics, etc.^26^. The microbial-mediated green production of AgNPs has recently been recognized as an interesting alternative to the physico-chemical synthesis^27^ because of their environmental-friendly production and purification. Microbial AgNPs synthesis is considered as a defense mechanism to the very reactive silver ions^28^. The *Marinomonas* sp ef1 AgNPs reported here showed high antimicrobial activity against twelve common nosocomial pathogens. Therefore, this synthesis capability in *Marinomonas* sp ef1 may serve to protect the bacterium from metal toxicity and pathogens. The ciliate may also receive the same benefits due to the long-term association. The antimicrobial activity represents also a promising tool to overcome the emergence of antibiotic-resistant pathogens and could serve as future adjuvant for conventional antibiotics.

## Materials and methods

### Strain isolation and growth conditions

*Marinomonas* sp. ef1 was isolated from *E. focardii* cells. *E. focardii* logarithmic growing cultures were harvested by centrifugation at 3000 rpm for 5 min. The pellet was resuspended with sterile sea water. The suspension was sonicated for 5–10 seconds at a pulse rate of 6 V to break the host cell cortex. The total cell extract was then inoculated directly into the Lysogeny Broth (LB: 1% tryptone, 0.5% yeast extract, 1% NaCl) with agar (1,5%) and incubated at 4 °C for one week. The pure single colonies were sub cultured routinely on LB agar plates. We obtained five discrete isolated strains that were stored in 30% glycerol at −20 °C for further use.

To estimate the optimal growing temperature in LB medium, cells were grown at 4 °C, 10 °C, 22 °C and 30 °C, and the increasing of the absorbance was monitored at 600 nm (OD600) using a spectrophotometer (Fig. S1). Growth data were finally fitted with a logistic standard model^29^.

### DNA extraction, 16S rDNA PCR amplification and phylogenetic analysis

Total genomic DNA were extracted from fresh overnight culture at 22 °C using PureLink^TM^ Genomic DNA mini kit (Invitrogen) according to manufacturer’s instructions. The quantity and quality of the extracted DNA were determined using the ND-1000 spectrophotometer (NanoDrop Technologies, Wilmington, DE) and by agarose (1%) gel electrophoresis. The 16S rRNA gene was amplified by PCR using bacterial universal degenerated primers 27 F (5′-AGAGTTTGATCMTGGCTCAG 3′) and 1492 R (5′-TACGGYTACCTTGTTACGACTT 3′), as forward and reverse primers, respectively. The amplification was performed in a Biometra Thermal Cycler (Biometra Ltd., Kent, UK) and was composed by an initial denaturation of 94 °C for 5 min, followed by 30 cycles of denaturation at 94 °C for 1 min, annealing at 60 °C for 1 min, and extension at 72 °C for 1 min. A final extension step was performed at 72 °C for 5 min. The Sanger sequencing of 16S rDNA amplicon was performed by the BMR Genomics (Padova, Italy). The full length of 16S rRNA gene sequence is deposited at the Genbank database (http://www.ncbi.nlm.nih.gov) under the acc. No: MF156139. The evolutionary history of the 16S rRNA genes was inferred by using the Maximum Likelihood method based on the Hasegawa-Kishino-Yano model^30^. The tree with the highest log likelihood (−5139.3684) is reported. Tree(s) for the heuristic search were obtained by applying the Neighbor-Joining method to a pairwise distances matrix estimated using the Maximum Composite Likelihood (MCL) approach. The analysis involved 13 nucleotide sequences. A total of 1805 positions were in the final dataset. hylogenetic analysis of *Marinomonas* sp ef1 open reading frames predicted from the genomic island was performed by using the Maximum Likelihood method and the JTT matrix-based model^31^. The tree with the highest log likelihood (−42791,8997) is reported. The percentage of trees in which the associated taxa clustered together is reported next to the branches. Tree(s) for the heuristic search were obtained by applying the Neighbor-Joining method to a pairwise distances matrix estimated using a JTT model. The analysis involved 83 amino acid sequences. A total of 1062 positions were in the final dataset. Evolutionary analyses were performed using MEGA5^32^.

### Whole genome sequencing, annotation and genomic island (GEI) identification

The genome was sequenced by Next Generation Sequencing (NGS) at BMR Genomics (Padova, Italy) using a Whole Genome Shotgun (WGS) approach, using the Illumina MiSeq v3 (300 bp paired-end) kit. Illumina reads were were merged using FLASH^33^ and *“de novo”* assembled using Newbler v.2.7^34^. The genome was annotated using Prokka version 1.11^35^, Blast2GO^36^ and covtobed^37^. The annotation step was performed with default parameters, and the whole assembly pipeline is included in the Metable project repository (https://github.com/quadram-institute-bioscience/metable_project). The annotation interface with BLAST server^38^, can be accessed at http://metable.seq.space. The WGS project is available at DDBJ/ENA/GenBank under the accession NHTT00000000.1. The version described in this paper is the NHTT00000000.1.

Genomic islands **(**GEI) were identified using Mauve^39^, a system for constructing multiple genome alignments. *Marinomonas* sp. ef1 genome was aligned with those from *M. polaris*, *M. acquimarina, M. mediterranea, Marinomonas* MWYL1, *M. profundimaris, M. ushuaiensis*. to produce a set of sequences found exclusively in *Marinomonas* sp. ef1 and a list of genome alignment coordinates that can be elaborated using a custom Perl script. GEIs were confirmed by estimation of different GC content and by a further BLASTn search on the other *Marinomonas* genomes.

Gene Ontology (GO) analysis was obtained by an enrichment evaluation using the Fisher’s Exact Test of the Blast2GO software^36^. The distributions of *Marinomonas* sp. ef1 and *M. polaris* CK13 GO terms were compared considering the following GO categories: molecular function, biological process and cellular component. A p-value threshold of 0.05 and a multiple testing correction of false discovery rate (FDR) as p-value filter mode were used^40^.

### AgNPs synthesis and purification

The bacterial biomass was obtained by inoculating *Marinomonas* sp ef1 into Luria-Bertani (LB) liquid medium (Tryptone, 10 g, Yeast extract, 5 g, NaCl, 10 g in 1 L of ddH_2_O). The flasks containing *Marinomonas* sp ef1 cultures were incubated on a water-bath set at 22 °C and 220 rpm, for 24 hours. The cultures were then centrifuged at 6000 g using a Beckman J2–21 for 30 minutes. After the supernatant removal, approximately 2 mg of bacterial biomass was suspended in a water solution containing 1 mM AgNO_3_ and transferred into an Erlenmeyer flask. The mixture was then placed again in the water-bath at 22 °C and at 200 rpm, for 24 h in bright conditions. A Erlenmeyer flask containing a heat killed *Marinomonas* sp ef1 culture containing 1 mM AgNO_3_ was maintained in parallel as negative control. The bioreduction of Ag^+^ ions was monitored by observing the changes of the bacterial biomass-AgNO_3_ mixture colors from white to dark brown and by UV–visible spectroscopy (Fig. S1). The UV–visible spectrum of a 0.1 ml aliquot diluted into 0.9 ml of ddH_2_O was recorded from 300 to 800 nm wavelengths at room temperature with a UV-1800, Shimadzu spectrophotometer, using ddH_2_O as blank.

For AgNPs purification, the bacterial biomass was collected by centrifuging the solution at 6000 g using a Beckman J2–21 for 30 min and suspended in ddH_2_O. The cells were ultra-sonicated at a pulse rate of 6 V for 30 seconds and 10 times. The solution was them centrifuged at 14,000 g on a Eppendorf 5417 centrifuge for 30 min and the supernatant was collected. To remove contaminating debris and proteins the resulting solution was filtered using Sephadex G-50 Medium and 10 mM Tris buffer (pH 7.0). AgNPs were finally recovered from the solution by adding 3 volumes of isopropyl alcohol, a non toxic solvent able to dissolve non-polar compounds. To obtain a purified NPs enriched powder, the mixture was incubated on the orbital shaker overnight to allow isopropyl alcohol evaporation.

### Chemical synthesis of silver nanoparticles

A 1 mM sodium citrate solution was added drop by drop to a boiling solution of 1 mM of AgNO_3_ until the solution turned into grayish-yellow color, indicating the formation of Ag+ ions. Heating was continued for 60 s, then the solution was cooled to room temperature.

### Scanning electron microscopy (SEM), Transmission electron microscopy (TEM), Energy Dispersive X-Ray Analysis (EDAX), Fourier transform infrared spectroscopy (FTIR), X- Ray Diffraction Analysis (XRD), Dynamic Light Scattering (DLS) and Zeta Potential Measurements

AgNPs size and shape characterization was carried out by SEM (ZIESSA, Sigma 300) analysis. Purified AgNPs were sonicated for 15 min to reach a uniform distribution. A drop of this solution was loaded on carbon-coated copper grids and evaporated under infrared light for 30 min.

TEM analysis was made on a TEM PHILIPS EM208S using an acceleration voltage of 100 kV. A drop of purified AgNPs was loaded on Nitrocellulose and Formvar coated copper TEM grids. After 2 min, the extra solution was removed and the grid was dried prior measurements. Data were analysed by a Statistical Software (StatSoft, USA) using the variability plot of average methods. The size distribution of AgNPs was estimated using TEM Imaging and Analysis software (TIA) on 100 measurements.

EDAX analysis was performed using a FESEM equipped with an EDAX attachment. For the FTIR analysis in attenuated total reflection (ATR), AgNPs were deposed on the single-reflection diamond element of the ATR device (Quest, Specac) and dried at room temperature. The ATR/FTIR spectrum was collected by the Varian 670-IR spectrometer, equipped with a nitrogen-cooled Mercury Cadmium Telluride detector. The following settings were employed: scan speed of 25 kHz, spectral resolution of 2 cm^−1^, 512 scan coadditions, and triangular apodization^41^.

For the X-ray Diffraction measurements, drop-coated films of AgNPs were prepared. A Philips PW 1830 bragg-Brentano diffractometer was used operating at 40 kV and 25 mA using Cu Kα radiation (λ = 1.5405 Å) monochromatised by means of a diffracted beam graphite crystal. Step scan XRD pattern was collected in the 2θ range from 10° to 80° with a 0.02° step and 3 s/point counting time. The XRD patterns were compared with the Joint Committee on Powder Diffraction Standards (JCPDS) library to determine the crystalline phases present.

Dynamic Light Scattering (DLS) and Zeta Potential Measurements were performed by using a Zetasizer Nano ZS (Malvern Instruments Ltd., UK). AgNPs particles sizing was based on the Mie-scattering method. Dynamic fluctuations of light scattering intensity caused by the Brownian motion of the particles was measured in triplicate with a temperature equilibration time of 1 min at 25 °C. High multi-modal resolution mode was set for data processing.

### Antibacterial activity of AgNPs by kirby–bauer disk diffusion method

The antibacterial activity of AgNPs was carried out using Kirby–Bauer Disk Diffusion Susceptibility Test method^42^ following the CLSI guidelines^43^ on twelve different clinical isolates. Gram positive Bacteria: *Staphylococcus aureus, Staphylococcus epidermidis, Streptococcus agalactiae*. Gram negative Bacteria: *Escherichia coli, Klebsiella pneumonia, Pseudomonas aeruginosa, Proteus mirabilis, Citrobacter koseri, Acinetobacter baumanii, Serratia marcescens*. Fungi: *Candida albicans, Candida parapsilosis*. The pathogenic cultures were subculture into Brain Heart Infusion (BHI) (Sharlab, Italy) broth and incubated at 37 °C to attain 10^5^–10^6^ CFU ml^−1^ and then adjusted to 0.5 McFarland turbidity. The bacteria strains were spread on Mueller-Hinton agar (MHA) (Merck, Germany) plates using sterile cotton swab. Sterile 6 mm diameter Whatman No. 1 filter paper disks were soaked with 25 μl of *Marinomonas* AgNPs, or chemically synthesized AgNPs, or AgNO_3_ solutions. The disks were then placed on the agar plates and incubated at 37 °C. The zone of inhibition was observed after 24 h of incubation and expressed in millimetres (zone of inhibition ± SD). Dose dependent antimicrobial activity was carried out using different concentrations of AgNPs or AgNO_3_, 0.4Mm, 0.8 mM, 1 mM, 2 mM, 3 mM, 4 mM and 6 mM on the MHA plates. All the experiments were done in triplicate, and the results were expressed as the mean ± SD.

### Minimum Inhibitory concentration (MIC) and minimum bactericidal concentration (MBC) evaluation

The MIC and MBC evaluation of *Marinomonas* synthesized AgNPs were performed using the method described in the CLSI guideline^43^.

The MIC test was performed against the microbial isolates reported above in a 96-well round bottom microtiter plate using the standard broth microdilution method. The pathogens cultures were adjusted to a concentration of 0.5 McFarland units. The *Marinomonas* AgNPs stock solution in sterilized deionized water was prepared by ultrasonication to reach the concentration of 200 μg/ml. AgNPs stock solution was serially diluted in BHI broth: in the first row 100 μl of NPs was added to 100 μl of BHI, then 100 μl form the first row was added to 100 μl of BHI of the second row, and so on. Finally, 100 μl was discarded such that the wells in first row of the microtiter plate contained the highest concentration of AgNPs, while the wells of the last row contained the lowest concentration. Then, 100 μl aliquot of McFarland standard suspension of bacteria was added in each well. A control containing only medium and AgNPs (K+) and another containing medium and the bacterial culture (K−) were added to the plates. The microtiter plate was then incubated at 37 °C for 24 h. The MIC value defined as the lowest concentration of antibacterial agents that inhibits the growth of bacteria.

The MBC test was performed on the MHA plates. The MBC was taken as the lowest concentration of the antibacterial agents that completely kill the bacteria. To check MBC the suspension from each well of microtiter plates was plated into MHA plate and were incubated at 37 °C for 24 h. MBC value was taken as the lowest concentration with no visible growth on the MHA plate. All the experiments were carried out in triplicate and the results were expressed as mean ± SD.

### *Marinomonas* AgNPs toxicity on Normal Human Dermal Fibroblasts (NHDF) cells

NHDF cells were grown to confluence in culture flasks in Dulbecco’s modified Eagle’s medium (DMEM) containing 10% heated inactivated fetal bovine serum (FBS, Gibco, USA) and 1% penicillin/streptomycin antibiotics (Sigma, USA). For all the experiment, cells were grown in a 5% CO_2_ humidified atmosphere incubator at 37 °C. Confluent fibroblast monolayers were propagated by trypsinization (0.1% trypsin and 0.02% EDTA).

AgNPs at different concentrations (100, 50, 25, 1, 0, 5 and 1 µg/ml) were placed into 96-well plates, and then sterilized under UV irradiation (254 nm, ≈7 mW cm^−2^) for 1 h. NHDF cells were then seeded on the AgNPs containing wells at a density of 10 × 10^3^ cells per well and incubated at 37 °C. After 24 and 48 h of incubation, the medium of each well was removed and replaced by a mixture of 100 μL of fresh culture medium and 20 μL of MTS/PMS (phenazine methosulfate) reagent solution and incubated for 4 h, at 37 °C. The absorbance of each sample was determined at 492 nm using a microplate reader (Bio-rad xMark microplate spectrophotometer). As control, cells incubated with ethanol (96%) (K+) and cells incubated only with culture medium (K−) were used. The half-maximal inhibitory concentration (IC50) values were calculated using the statistic software GraphPad Prism 5 (USA). The effect of AgNPs on cellular adherence and morphology was observed under inverted light microscope (Nikon TS100). Cellular confluence percentage was determined using the ImageJ 1.49 processing software (National Institute of Health, Maryland, USA).

### Statistical analysis

To determine statistical significance, One-way analysis of variance (ANOVA) with Tukey’s multiple comparison test was performed using Graphpad Prism software (La Jolla, CA, USA).

## Results

### Identification of the *Marinomonas* strain isolated from *E. focardii* cells and phylogenetic analysis

Following the isolation protocol described under Material and Methods, we obtained five discrete single bacterial colonies. A preliminary characterization of these new strains was obtained by the 16S rDNA analysis. The sequence of a 1450 bp PCR amplicons from each colony was used as query for a BLASTn search in NCBI (https://blast.ncbi.nlm.nih.gov/Blast.cgi). One of the amplicon corresponded to a new *Pseudomonas* strain^44,45^. The highest similarities of a second amplicon sequence were found in some Antarctic *Marinomonas* strains including the *Marinomonas* Bsw10506 (99% identical, E-value = 0.0), a marine planktonic bacterium isolated from Antarctic seawater, and *M. polaris* CK13 (99% identical, E-value = 0.0), isolated from coastal sea water off the sub Antarctic Kerguelen islands^27^. We named the newly isolated strain *Marinomonas* sp. ef1. The other strains are still under analysis.

**Figure 1:**
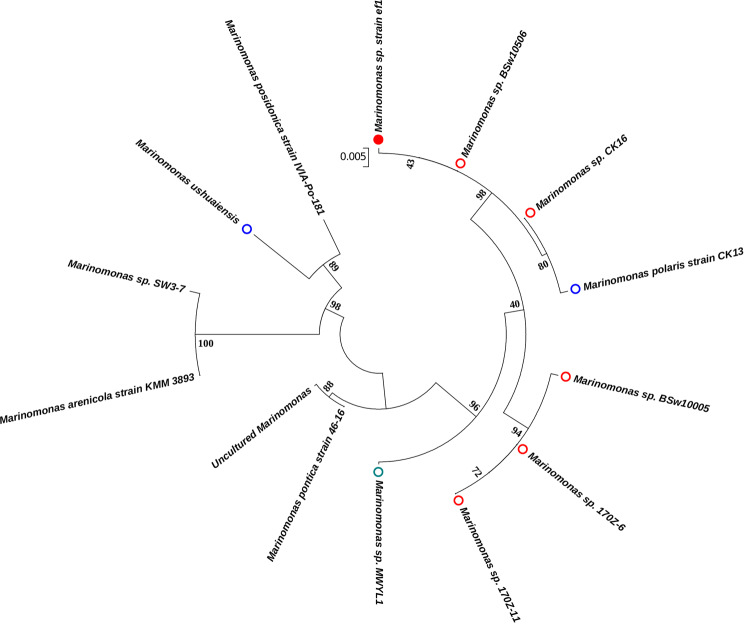
Phylogenetic analysis of *Marinomonas* species based on 16S rRNA gene sequences. *Marinomonas* sp. ef1 is evidenced by a red full circle; Antarctic and sub antarctic *Marinomonas* are evidenced by empty red and blue circles, respectively. *Marinomonas* sp. MWYL1, the closest relative with a published whole-genome sequence, is indicated by a green empty circle. Acc nos: *Marinomonas* sp.ef1: MF156139.1; *Marinomonas* sp. BSw10506: EF437161.1; *Marinomonas* sp. MWYL1; *Marinomonas* sp. 170Z-6; *Marinomonas polaris* strain CK13: *Marinomonas* sp. CK16: NR_042340.1; *Marinomonas ushuaiensis*: AJ627909.1; *Marinomonas posidonica* strain IVIA-Po-181: NR_074719.1; *Marinomonas* sp.SW3–7: FR744845.1; *Marinomonas pontica* strain 46–16: NR_042965.1; *Marinomonas arenicola*: NR_112826.1; Uncultured *Marinomonas*: KX014047.1.

The phylogenetic tree based on the 16S rDNA (Fig. 1) places *Marinomonas* sp. ef1 (evidenced by a red full circle) as the sister group of *Marinomonas* Bsw10506, *M. polaris* CK13 and *Marinomonas* sp. CK16 (this latter also isolated from Antarctica seawater), confirming its evolutionary relationship between these Antarctic and sub Antarctic *Marinomonas* strains. In general, Antarctic and sub Antarctic *Marinomonas* (Fig. 1, empty red and blue circles, respectively) form two main clusters in a single clade with the exception of *Marinomonas ushuaiensis* isolated from the Ushuaia seawater, located at the southernmost tip of Argentina and also considered as a sub Antarctic species. These results suggest that the *Marinomonas* of the Antarctic lineage evolved from a single ancestor, whereas the sub Antarctic *Marinomonas* may not share the same ancestry.

### Genome analysis

**Figure 2:**
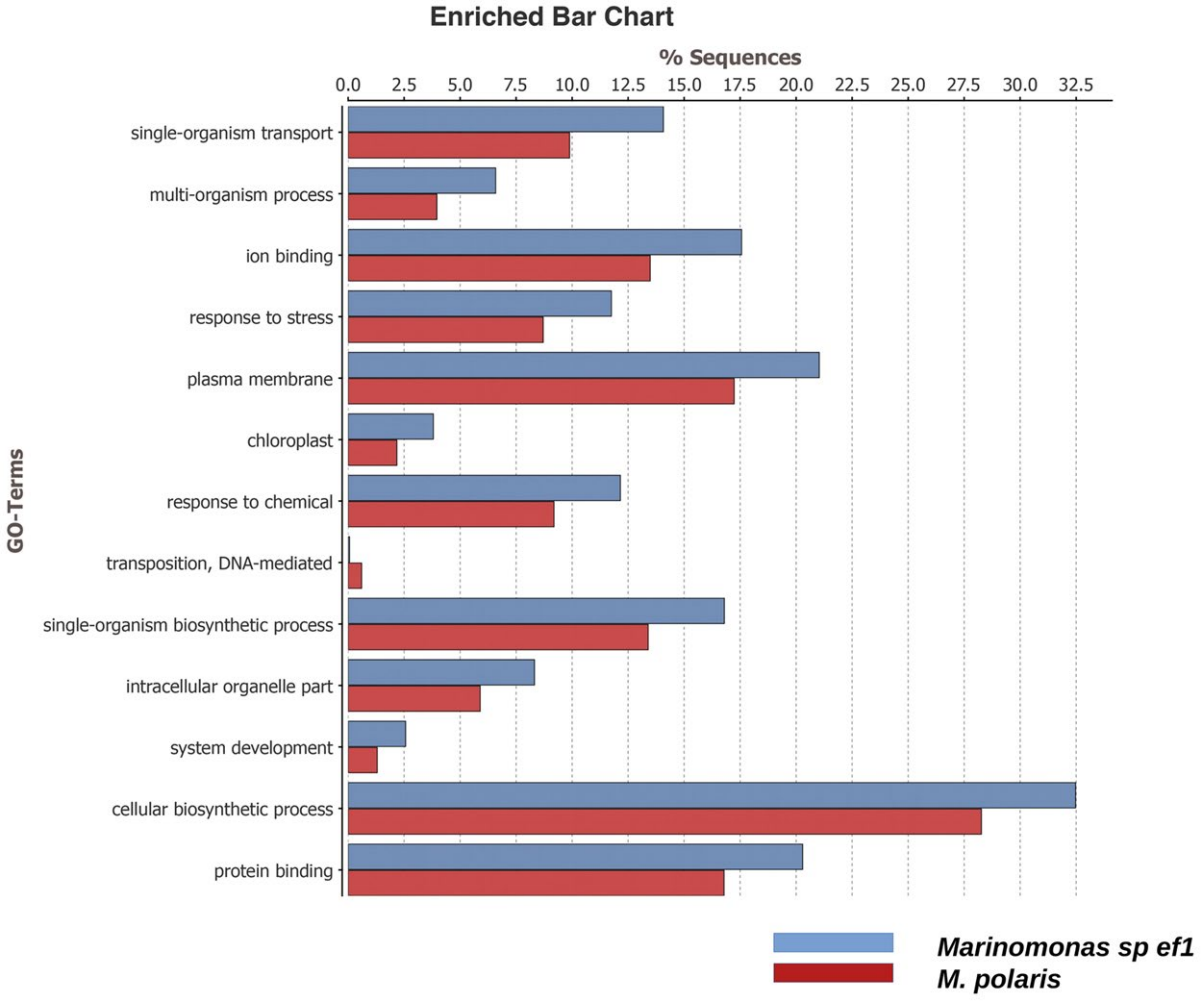
Enrichment analysis of the Gene Ontology terms of *Marinomonas* sp. ef1 (blue bar) and *M. polaris* (red bar) using the Fisher’s Exact Test available in the Blast2GO software.

*Marinomonas sp. ef1* whole genome consists of 4,740,116 bp and 4343 coding sequences. Table 1 reports *Marinomonas* sp. ef1 genome characteristics (length of the contigs, G + C content, number of coding sequences, etc) compared with *M. polaris* CK13, *Marinomonas* MWYL1 and *M. ushuaiensis* (i.e. the closest relatives with a published whole-genome sequence; evidenced by a green and blu empty circles, respectively, in Fig. 1). The genome characteristics of the three *Marinomonas* are similar. To verify gene loss in the associated bacterial strain, we performed a gene ontology (GO) and an enrichment analysis using the Fisher’s Exact Test^34^. Then, we compared term changes with respect to *M. polaris* CK13, the evolutionary closest free-living species with a published whole-genome sequence (Fig. 2). The comparison shows that the only less represented *Marinomonas* sp. ef1 GO term is “transposition DNA mediated”. The detailed list of the transposase genes in *Marinomonas* sp. ef1 and *M. polaris* CK13 is reported in Table S1.

**Table 1:** *Marinomonas* sp. ef1 genome characteristics.

Features	*Marinomonas* sp. ef1	*M. polaris*	*M. ushuaiensis*	*M. sp. MWYL1*
Contigs	40	52	39	1
Size (bp)	4740116	5038537	3342098	5100344
G + C content (%)	42.55	42.47	41.10	42.60
Number of CDSs*	4343	4605	3055	4541
Total CDSs size (bp)	4205577	4466406	2961891	4517497
Coding %	88.72%	88.64%	88.62%	88.57%
Average CDS length (nt)	966	969	969	995
tRNAs	84	58	58	83
tmRNA	1	1	1	1
rRNA genes	6	13	5	25
Number of genes with assigned function	3801 (87.52% of CDSs)	3990 (86.64% of CDSs)	2420 (79.21% of CDSs)	3932 (86.59% of CDSs)
Number of genes without assigned function	542	615	635	609
Number of predicted enzymes	1109 (25.53% of CDSs)	1047 (22.74% of CDSs)	1079 (35.32% of CDSs)	1369 (30.15% of CDSs)

*CDS: Coding sequence.

**Figure 3:**
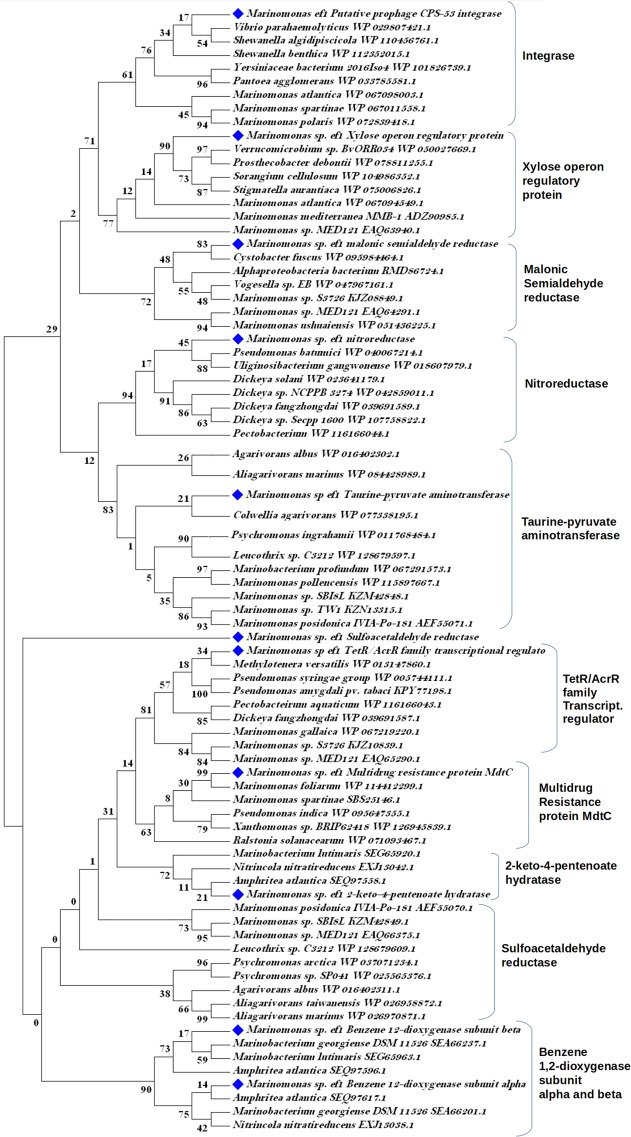
Neighbour Joining phylogenetic analysis of *Marinomonas* sp ef1 open reading frames predicted from the genomic islands. The blue diamonds evidence the sequences belonging to *Marinomonas* sp. ef1.

To obtain a set of genomic islands (GEIs) found exclusively in *Marinomonas* sp. ef1, the enriched terms were also compared with genomes from the following *Marinomonas* strains: *M. polaris*, *M. acquimarina, M. mediterranea, Marinomonas* MWYL1, *M. profundimaris, M. ushuaiensis*. We characterized five GEIs, from which we predicted twelve open reading frames (ORFs) (Table S2). To confirm the horizontal gene transfer (HGT) origin of these sequences we constructed a phylogenetic tree based to these ORFs and their homologous identified in the databank (Fig. 3A,B): with the only exception of the multidrug resistance protein MdtC, all the *Marinomonas sp*. ef1 sequences clustered in different clades from those containing the other *Marinomonas s*equences. It is interesting to note the tree position of the *Marinomonas sp*. ef1 sulfoacetaldehyde reductase that is completely separated from the clade containing the homologous sequences from the other bacteria, suggesting a very ancient lateral gene acquisition and subsequent sequence modifications. The ORF coding for the putative prophage CPS-53 integrase most probably derived from a bacteriophage infection. Three of the other identified ORFs encode for enzymes that are not present in other *Marinomonas* strains, such as 2-keto-4-pentenoate hydratase, putative NAD(P)H nitroreductase YfkO, and benzene 1,2-dioxygenase subunit alpha and beta. These laterally acquired sequences may confer unique metabolic properties to *Marinomonas* sp. ef1.

From this analysis it is not possible to identify the “donors” of the horizontally transferred genes: only five of them branched from a single sister taxon and only two (the 2-keto-4-pentenoate hydratase and the benzene 1,2-dioxygenase subunit alpha) share a common ancestor with *Amphritea atlantica* even though the bootstrap values are too low to confirm the ancestry. Additional bacterial genomic sequences are necessary for a deeper understanding of this phenomenon.

### Bioconversion of silver nitrate into silver nanoparticles

**Figure 4:**
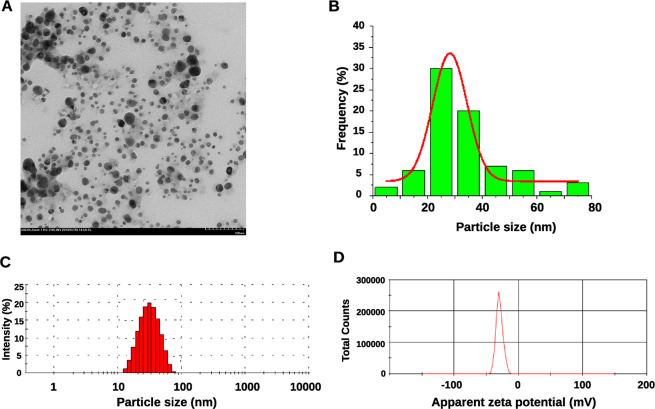
Morphological characterization of *Marinomonas* sp. ef1 AgNPs. (**A**) Transmission Electron Microscopy (TEM) image. Bar: 100 nm. (**B**) AgNP size distribution estimated from randomly selected AgNPs on the TEM image. (**C**) Dynamic light scattering (DLS) analysis. The red histograms show the particle size of the AgNPs ranging from 20 to 70 nm. The estimated AgNPs average size is 40  ±  2.4 nm. (**D**) Zeta potential distribution estimation: the apparent zeta potential is −27.1 ± 0.6 mV.

Two horizontally acquired ORFs, the NAD(P)H nitroreductase YfkO and the putative malonic semialdehyde reductase RutE (nitroreductase FMN family), were involved in the reduction of nitrogen containing compounds. Furthermore, the NAD(P)H nitroreductase YfkO has been reported be responsible for the bioreduction of silver ions in the AgNPs formation^26,46^, a process never described for *Marinomonas* strains. We investigated the ability of *Marinomonas* sp. ef1 to synthesize AgNPs by incubating the bacterial biomass in a solution containing 1 mM of AgNO_3_, at 22 °C (Fig. S2 and Table S1). A color change from white to brown occurred within 24 h of incubation in the presence of light (Fig. S2). The brown color was maintained throughout the 72 h observation period. No color changes were observed in the experimental control culture containing heat-killed bacterial biomass and silver nitrate (not shown). *Marinomonas* sp. ef1 AgNPs formation was confirmed by UV-vis spectroscopy. We observed a strong peak located at 430 nm (Fig. S1). The presence of a single SPR peak suggested a spherical shape. Transmission Electron Microscopy (TEM) was used to determine the size and shape. Aliquots of AgNPs in solution were placed onto a nitrocellulose/Formvar coated copper grid and allowed to dry under ambient conditions. TEM micrographs showed that the particles were of spherical shape, well separated and not in direct contact with each other even when these formed aggregates (Fig. 4A), suggesting the presence of capping peptides around each particle, probably playing the role of nanoparticle stabilization. To estimate particle size and size distribution, 200 AgNPs obtained from TEM images were randomly selected and evaluated using Image J 1.45 s software. *Marinomonas* AgNPs were in the range of 10–80 nm and the average size was about 30.02 ± 13.41 nm (Fig. 4B).

The size distribution profile of the *Marinomonas* sp. ef1 AgNPs was better evaluated by Dynamic Light Scattering (DLS) (Fig. 4C). We found that the AgNPs size ranges from 20 to 80 nm. The average size is about 40  ±  2.4 nm with a Polydispersity Index (PdI) value of 0.201. Zeta potential measurement indicates that the AgNPs show negative charge of −27.1 ± 0.6 mV (Fig. 4D), that explains their polydispersed nature^47,48^. The single peak that was obtained from this analysis indicates a good quality of the *Marinomonas* sp. ef1 AgNPs.

### Chemical composition of *Marinomonas* sp ef1 AgNPs

**Figure 5:**
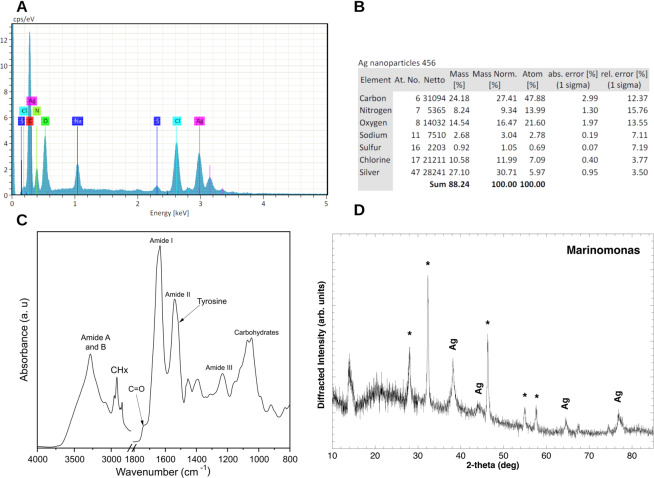
(**A**,**B**) EDAX analysis of *Marinomonas* sp ef1 AgNPs. Ag, C, N and O indicate the silver (the highest peak, recorded at at 3 keV), carbon, nitrogen and oxygen signals. (**C**) Fourier transform infrared (FTIR) spectrum of *Marinomonas sp*. ef1 AgNPs. The assignment of selected bands is shown: the peaks around 3282 cm^−1^ (Amide A) and 3070 cm^−1^ (Amide B) were mainly assigned to the NH vibrations. The Amide I band maximum absorption, due to the C = O stretching of the peptide bond, occurred around 1633 cm^−1^. The Amide II band, due to the amide NH bending, peaked around 1539 cm^−1^. The complex absorption in the 1200-950 cm^−1^ spectral region could be tentatively assigned to carbohydrate absorption. The 1740 cm^−1^ peak was characteristic of C = O carbonyl groups. (**D**) XRD spectra of the *Marinomonas* sp. ef1 AgNPs: the diffraction pattern of the *Marinomonas* sample displays four peaks (at 2-theta angles of at 38.95°, 45.12°, 65.39° and 78.12°, corresponding to the diffraction of (1 1 1), (2 0 0), (2 2 0) and (3 1 1) lattice planes respectively) typical of the cubic form of silver.

With the Energy Dispersive X-ray (EDX) spectrum analysis (Fig. 5A,B), we observed an intense Ag signal at 3 keV. AgNPs typically show a strong signal peak at 3 keV, due to surface plasmon resonance. However, other elements (C, N and O) were recorded at normal mode (Fig. 5A,B). These elements probably derive from the emissions of the capping proteins.

To confirm possible interactions between silver salts and capping proteins, we performed Fourier transform infrared (FTIR) measurements (Fig. 5C). The amide linkages between amino acids residues in proteins give origin to well-known signatures in the mid-infrared spectral region^49^.

The FTIR spectrum was characterized by the protein Amide bands (Fig. 5C, Table 2). In particular, the peaks around 3282 cm^−1^ (Amide A) and 3070 cm^−1^ (Amide B) were mainly assigned to the NH vibrations. The Amide I band maximum absorption, due to the C = O stretching of the peptide bond, occurred around 1633 cm^−1^. The Amide II band, mainly due to the amide NH bending, peaked around 1539 cm^−1^ ^49^. The complex absorption in the 1200-900 cm^−1^ spectral region could be tentatively assigned to polysaccharides and nucleic acids absorption. Finally, the 1740 cm^−1^ peak was characteristic for the C = O carbonyl groups^50,51^. The overall spectral features (Fig. 5C) unambiguously showed the presence of polypeptides in the biosynthesized AgNPs, as demonstrated by the high intensities of the Amide bands. As reported in Fig. 5C and Table 2, the FTIR analysis also indicated the presence of other biomolecules and in particular of polysaccharides and nucleic acids (IR bands in the 1200-900 cm^−1^ spectral region).

**Table 2:** Tentative assignment of some bands observed in the FTIR spectra of *Marinomonas sp*. ef1 AgNPs^49–51^.

Peak position (cm^−1^)	Assignment
~3500	−OH group
3282	−NH (Amide A)
3070	−NH (Amide B)
3000-2800	−CH3 and -CH2 stretch of aliphatic compounds
1740	C=O, esters
1633	C=O, Amide I
1539	NH, Amide II
1454	CH_2_ methylene
1391	COO^−^group in carboxylic acid
1250-1220	PO^2-^; Amide III
1200-900	C–O–C, C–O dominated by ring vibrations of carbohydrates; C–O–P; P–O–P; PO^2−^

The presence of metallic silver nanoparticles was confirmed by XRD analysis (Fig. 5D). The diffraction pattern of the *Marinomonas* sample displays four peaks (at 2-theta angles of at 38.95°, 45.12°, 65.39° and 78.12°, corresponding to the diffraction of (1 1 1), (2 0 0), (2 2 0) and (3 1 1) lattice planes respectively) typical of the cubic form of silver. Their large full width at half maximum indicates a low crystallinity of metallic silver (i.e. the silver is present in the form of nanoparticles). Furthermore, additional sharp peaks are present (indicated by asterisk in Fig. 5D) which indicate the presence of crystalline AgCl. The presence of AgCl peaks may be due to the reaction of the silver present in solution with the chloride of the culturing medium.

### Antibacterial activity of *Marinomonas* sp. ef1 AgNPs

We tested the antibacterial activity of Marinomonas sp. ef1 AgNPs against twelve pathogenic microorganisms: Staphylococcus aureus, Staphylococcus epidermidis, Streptococcus agalactie, Escherichia coli, Klebsiella pneumonia, Pseudomonas aeruginosa, Proteus mirabilis, Citrobacter koseri, Acinetobacter baumanii, Serratia marcescens, Candida albicans, Candida parapsilosis. We compared this activity with that of AgNO_3._ A concentration gradient (0.4 mM, 0.8 mM, 1 mM, 2 mM, 3 mM, 4 mM and 6 mM) was tested for both AgNPs and AgNO_3_. A volume of 25 µl of both AgNPs and AgNO_3_ was spotted in the paper disk (Fig. S3). Increasing concentrations of both AgNPs and AgNO_3_ enlarged the inhibition halo (Fig. S3). However, AgNPs showed stronger antibacterial effect than AgNO_3_ (Table 3). Among Gram- bacteria the larger inhibition zone at 6 mM of AgNPs was obtained against Escherichia coli (Ø 21.0 mm) and the lowest against Serratia marcescens (Ø 18.0 mm). Among Gram+ bacteria the highest inhibition zone was obtained against Staphylococcus aureus (Ø 21.0 mm). Among fungi, the larger zone was obtained against Candida albicans (Ø 20.0 mm). It is interesting to note that Marinomonas AgNPs activity is similar or much higher than the antibiotics used as a control, i.e Ampicillin or Amphotericin b, in particular against Citrobacter koseri and Acinetobacter baumanii (Table 3 and Table S4).

**Table 3:** Dose dependent antimicrobial activity of *Marinomonas* sp. ef1 AgNps against various pathogenic microorganisms.

S.no	*Microorganisms*	0.4 mM			0.8 mM			1 mM			2 mM			3 mM			4 mM			6 mM			
		C^1^	T^2^	IZS^3^ (T-C)	C^1^	T^2^	IZS^3^ (T-C)	C^1^	T^2^	IZS^3^ (T-C)	C^1^	T^2^	IZS^3^ (T-C)	C^1^	T^2^	IZS^3^ (T-C)	C^1^	T^2^	IZS^3^ (T-C)	C^1^	C^2^	T^2^	IZS^3^ (T-C)
***Gram positive bacteria***																							
1	*Staphylococcus aureus*	9 ± 0.2	11 ± 0.4	2 ± 0.2	10 ± 0.2	13 ± 0.3	3 ± 0.1	8 ± 0.3	15 ± 0.4	7 ± 0.1	12 ± 0.3	17 ± 0.4	5 ± 0.2	13 ± 0.2	18 ± 0.2	5 ± 0.3	14 ± 0.3	19 ± 0.2	5 ± 0.2	16 ± 0.4	18 ± 0.2	21 ± 0.3	5 ± 0.2
2	*Staphylococcus epidermidis*	7 ± 0.3	9 ± 0.2	2 ± 0.3	8 ± 0.1	11 ± 0.2	3 ± 0.2	9 ± 0.4	13 ± 0.3	4 ± 0.2	10 ± 0.2	14 ± 0.3	4 ± 0.1	11 ± 0.3	15 ± 0.4	4 ± 0.2	12 ± 0.4	16 ± 0.2	4 ± 0.2	14 ± 0.3	22 ± 0.1	17 ± 0.4	3 ± 0.1
3	*Streptococcus agalactie*	8 ± 0.1	11 ± 0.2	3 ± 0.2	9 ± 0.3	13 ± 0.3	4 ± 0.2	8 ± 0.2	14 ± 0.1	6 ± 0.2	11 ± 0.4	15 ± 0.3	4 ± 0.3	13 ± 0.2	16 ± 0.3	3 ± 0.1	14 ± 0.3	17 ± 0.4	3 ± 0.3	16 ± 0.4	13 ± 0.2	19 ± 0.2	3 ± 0.2
***Gram negative bacteria***																							
4	*Escherichia coli*	8 ± 0.2	11 ± 0.3	3 ± 0.2	10 ± 0.3	16 ± 0.6	6 ± 0.1	11 ± 0.2	17 ± 0.3	6 ± 0.2	12 ± 0.5	18 ± 0.4	6 ± 0.2	13 ± 0.5	19 ± 0.2	6 ± 0.3	14 ± 0.2	19.5 ± 0.2	5.5 ± 0.2	16 ± 0.1	10 ± 0.1	21 ± 0.3	6 ± 0.1
5	*Klebsiella pneumoniae*	8 ± 0.3	12 ± 0.5	4 ± 0.2	10 ± 0.2	15 ± 0.4	5 ± 0.2	8 ± 0.4	16 ± 0.1	8 ± 0.2	12 ± 0.8	17 ± 0.2	5 ± 0.4	13 ± 0.2	18 ± 0.5	5 ± 0.2	14 ± 0.4	19 ± 0.3	5 ± 0.1	16 ± 0.2	12 ± 0.1	20 ± 0.2	4 ± 0.2
6	*Pseudomonas aeruginosa*	7 ± 0.4	13 ± 0.2	6 ± 0.3	9 ± 0.2	14 ± 0.3	5 ± 0.1	9 ± 0.1	15 ± 0.4	6 ± 0.1	12 ± 0.5	16 ± 0.2	4 ± 0.3	13 ± 0.2	17 ± 0.4	4 ± 0.2	13.5 ± 0.2	18 ± 0.3	5.5 ± 0.1	15 ± 0.3	18 ± 0.2	19 ± 0.5	4 ± 0.1
7	*Proteus mirabilis*	5 ± 0.2	12 ± 0.3	7 ± 0.2	6 ± 0.2	13 ± 0.5	7 ± 0.2	8 ± 0.2	14 ± 0.3	6 ± 0.2	10 ± 0.2	15 ± 0.3	5 ± 0.2	11 ± 0.2	16 ± 0.3	5 ± 0.0	12 ± 0.2	17 ± 0.5	5 ± 0.3	14 ± 0.2	14 ± 0.3	20 ± 0.4	6 ± 0.2
8	*Citrobacter koseri*	9 ± 0.4	11 ± 0.3	2 ± 0.3	10 ± 0.4	12 ± 0.2	2 ± 0.2	10 ± 0.4	15 ± 0.2	5 ± 0.2	12 ± 0.2	17 ± 0.3	5 ± 0.3	13 ± 0.3	18 ± 0.4	5 ± 0.3	15 ± 0.4	19 ± 0.4	3 ± 0.1	17 ± 0.3	R	21 ± 0.4	2 ± 0.4
9	*Acinetobacter baumanii*	10 ± 0.4	11 ± 0.2	1 ± 0.2	11 ± 0.3	12 ± 0.2	1 ± 0.3	10 ± 0.3	14 ± 0.4	4 ± 0.3	13 ± 05	15 ± 0.4	2 ± 0.2	14 ± 0.1	16 ± 0.3	2 ± 0.1	15 ± 0.3	18 ± 0.5	3 ± 0.1	17 ± 0.3	R	20 ± 0.2	3 ± 0.3
10	*Serratia marcescens*	7 ± 0.3	11 ± 0.1	4 ± 0.1	8 ± 0.1	12 ± 0.3	4 ± 0.1	9 ± 0.2	14 ± 0.3	5 ± 0.2	10 ± 0.2	15 ± 0.3	5 ± 0.2	12 ± 0.3	15 ± 0.2	3 ± 0.2	13 ± 0.3	16 ± 0.4	3 ± 0.1	14 ± 0.2	15 ± 0.3	18 ± 0.3	4 ± 0.2
***Fungi***																							
11	*Candida albicans*	7 ± 0.2	10 ± 0.3	3 ± 0.0	8 ± 0.2	12 ± 0.3	4 ± 0.2	8 ± 0.2	14 ± 0.4	6 ± 0.2	10 ± 0.6	16 ± 0.4	6 ± 0.2	11 ± 0.1	18 ± 0.1	7 ± 0.1	12 ± 0.2	20 ± 0.5	8 ± 0.3	14 ± 0.2	12 ± 0.2	20 ± 0.3	6 ± 0.1
12	*Candida parapsilosis*	7 ± 0.2	8 ± 0.4	1 ± 0.2	9 ± 0.3	11 ± 0.2	2 ± 0.3	8 ± 0.2	12 ± 0.2	4 ± 0.1	11 ± 0.3	14 ± 0.2	3 ± 0.3	12 ± 0.3	16 ± 0.4	4 ± 0.2	13 ± 0.1	17 ± 0.3	4 ± 0.3	15 ± 0.6	16 ± 0.1	19 ± 0.1	4 ± 0.

C^1^ = Control (AgNO_3_); C^2^ = control (ampicillin for bacteria, amphotericin B for *Candida*); T^2^ = Test (AgNPs); IZS^3^ = Increased zone size, obtained from the difference between the mm halo of the Control and the Test. Data were measured in mm and represent the mean of three experimental values.

MIC and MBC values of *Marinomonas* AgNps are reported in Table 4 and Fig. S3. The lowest MIC value of 3.12 μg/ml was found against *Proteus mirabilis*, whereas the highest MIC value of 12.5 μg/ml was found against *Escherichia coli*, *Klebsiella pneumoniae, Acinetobacter baumanii* and *Staphylococcus aureus*. Fungi appear more resistant to *Marinomonas* AgNps: the MIC value against *Candida parapsilosis* is 12.5 μg/ml, whereas against *Candida albicans* is 25 μg/ml.

**Table 4:** MIC and MBC of Marinomonas sp. ef1 AgNps against various pathogenic microbes.

Microbes	MIC µg/ml	MBC µg/ml
*Staphylococcus aureus*	12.5 ± 0.3	25 ± 0.2
*Escherichia coli*	12.5 ± 0.2	25 ± 0.1
*Klebsiella pneumoniae*	12.5 ± 0.3	25 ± 0.3
*Pseudomonas sp*	6.25 ± 0.1	12.5 ± 0.2
*Proteus mirabilis*	3.12 ± 0.1	6.25 ± 0.1
*Citrobacter koseri*	6.25 ± 0.2	6.25 ± 0.1
*Acinetobacter baumanii*	12.5 ± 0.2	12.5 ± 0.3
*Serratia marcescens*	6.25 ± 0.3	12.5 ± 0.2
*Candida albicans*	25 ± 0.5	25 ± 0.2
*Candida parapsilosis*	12.5 ± 0.3	25 ± 0.3

The lowest MBC values were observed for *Proteus mirabilis* and *Citrobacter koseri* (6.25 μg/ml). The highest values were recorded for *Escherichia coli*, *Klebsiella pneumonia*, *Staphylococcus aureus* and both *Candida* (25 μg/ml).

Finally, we also compared *Marinomonas* AgNPs antibacterial activity with that of chemically synthesized AgNPs and ddH_2_O and found that the formers are more active against the twelve pathogenic microorganisms described above (Fig. S4 and Table S3).

### *Marinomonas* AgNPs toxicity to mammalian cells

**Figure 6:**
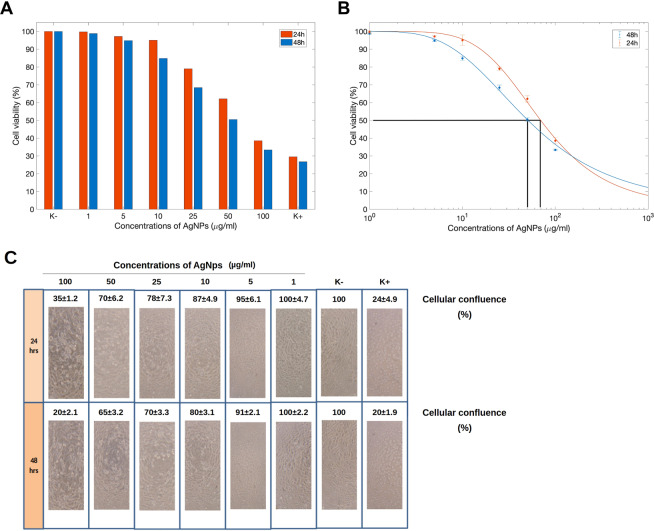
(**A**) Viability of Normal Human Dermal Fibroblasts (NHDF) cells after treatment with increasing concentration of *Marinomonas* AgNPs (from 1 to 100 µg/ml) for 24 and 48 hours evaluated by the MTT reduction assay. (**B**) Estimation of the IC50 values calculated on the basis of MTT reduction assay. (**C**) Cellular confluence estimation of NHDF after incubation with *Marinomonas* AgNPs. Representative images were taken using a Nikon TS100 inverted light microscope.

We evaluated *Marinomonas* AgNPs toxicity to Normal Human Dermal Fibroblasts (NHDF) by using the MTT ((3-(4,5-Dimethylthiazol-2-yl)-2,5-Diphenyltetrazolium Bromide) reduction assay. We estimated NHDF viability after 24 and 48 hours of incubation with increasing *Marinomonas* AgNPs concentration (from 1 to 100 µg/ml). We compared NHDF viability with that of untreated cells (K-, considered as 100% cell viability) and with that of NHDF cells treated with 96% ethanol (K+). After 24 hours, NHDF cell viability was affected by *Marinomonas* AgNPs only at concentrations of 25 µg/ml (79.01 ± 2.06% of viability), 50 (62.09 ± 3.70%) and 100 µg/ml (38.54 ± 3.70%) (Fig. 6A). However, NHDF viability was higher than that of cells treated with 96% of ethanol. By contrast, there was almost no cytotoxic effect observed at concentrations of 1, 5 and 10 µg/ml (99.78 ± 1.94%, 97.21 ± 0.84% and 95.07 ± 6.15% of cell viability, respectively). After 48 hours, a further reduction in cell viability was observed: in particular AgNPs at a concentration 10 µg/ml reduced NHDF viability to 84.88 ± 2.62%. The IC50 values resulted to be ~60 µg/ml and ~50 µg/ml after 24 and 48 hours of incubation, respectively (Fig. 6B).

Cellular monolayer confluence analysis on an inverted phase contrast microscope, performed 24 and 48 hours of incubation with *Marinomonas* AgNPs, confirmed the result reported above (Fig. 6C). After 24 hours, *Marinomonas* AgNPs at concentrations of 50 and 100 µg/ml interfere with NHDF capacity to adhere to the plate. By contrast, there was no significant effects on NHDF when AgNPs were used at concentrations of 1, 5 and 10 µg/ml. After 48 hours, a reduction in monolayer integrity to 91 ± 2.1% and 80 ± 3.1% was observed also in NHDF treated with 5 and 10 µg/ml of *Marinomonas* AgNPs, respectively. The concentration of 1 µg/ml did not affect the monolayer confluence, which was similar to that of untreated cells.

## Discussion

To our knowledge, *Marinomonas* sp. ef1 is the first *Marinomonas* strain isolated from a bacterial consortium associated to an Antarctic ciliate. The phylogenetic analysis of the 16S rDNA sequence indicates a close relationship with other free living Antarctic and sub Antarctic *Marinomonas* species. This evidence suggests that the association between *Marinomonas* sp. ef1 and the ciliate is a long-term interaction that started after the geographical isolation of the Antarctic continent from Gondwanaland and the formation of the Polar Front, dated over the last 60 mya^52^.

The genomic analysis revealed a reduced gene loss limited to sequences encoding for transposases. Genome reduction is seen as a hallmark for obligate endosymbiosis^53^ and it is considered the result of the coevolution between endosymbiont and host or the bottleneck of a small population size^54^. Limited signs of genome reduction were also reported for facultative endohyphal bacteria of diverse Ascomycota^55^. Considering that the symbiotic relationship between *Marinomonas* sp. ef1 and *E. focardii* appears not obligate and the *Marinomonas* sp. ef1 can survive outside the host in the presence of a carbon source, the same explanation may be proposed for the small reduction of *Marinomonas* sp. ef1 genome. We may also hypothesize that the maintenance of the entire bacterial metabolic potentiality is a “*nothing to be wasted*” strategy to survive in extreme and harsh conditions.

The *Marinomonas* sp. ef1 genome bioinformatic analysis identified five GEIs encoding for enzymes involved in metabolic pathways, such as 2-keto-4-pentenoate hydratase, benzene 1,2-dioxygenase alpha and beta subunits, and putative NAD(P)H nitroreductase YfkO. The phylogenetic analysis supports the hypothesis that these genes were acquired by horizontal gene transfer (HGT), providing an additional example that this is a major ongoing force in prokaryotic evolution and environmental adaptation^56^. However, the tree does not allow to identify the GEIs donor: only five of them branched from a single sister taxon represented by *Amphritea atlantica, Methylotenera versatilis*, *Cystobacter fuscus*, and *Colwellia agarivorans*. However, in most of the cases the bootstrap value was too low to confirm the ancestry. Three of the identified ORFs encoding for enzymes absent in other *Marinomonas* strains (2-keto-4-pentenoate hydratase, putative NAD(P)H nitroreductase YfkO and benzene 1,2-dioxygenase subunit alpha and beta) could confer unique metabolic properties, such as reduction of nitrogen-containing compounds, to *Marinomonas* sp. ef1. The acquisition of genes implicated in alternative metabolic pathways could be a strategy to survive in an environment poor of nutrients, such as the Antarctic seawater.

*Marinomonas* sp. ef1 is able to convert AgNO_3_ into AgNPs. AgNO_3_ is a toxic and corrosive compound. In general, the conversion of silver salts in NPs is a recognized mechanism of cell defense against metals^28^. Therefore, AgNPs could be used to neutralize toxic metals and to protect the cells from pathogens (see below) providing advantages to both the bacterium and the ciliate host. The average dimension of the *Marinomonas* sp. ef1 synthesized AgNPs is 40 nm. Our analyses confirm the presence of capping proteins in a not aggregated shape. *Marinomonas* sp. ef1 AgNPs characteristics appear similar to those produced by *Pseudomonas spp*^45,57,58^, even though Pseudomonas putida AgNPs were monodispersed and smaller in size (6 to 16 nm)^59^. *Marinomonas* sp. ef1 AgNPs show high antimicrobial activity and a low toxicity to mammalian cells. AgNPs antimicrobial activity remains to be understood. Several studies propose that AgNPs affect cell membrane integrity, disturbing permeability and respiration functions^60,61^. For this reason smaller AgNPs are more effective than larger ones because these possess large surface area available for interaction. It has been also proposed that AgNPs may enter inside the target cell^61^, or release silver from the NPs^62^.

This is the first report of AgNPs produced by a *Marinomonas* strain. The use of the biosynthesized AgNPs represents a promising approach to solve bacterial resistance. Alternative antimicrobial molecules can play a key role in pharmacotherapeutics^63,64^.

## Supplementary information

Supplementary information
