# Supplementary material for: Horizontal gene transfer and silver nanoparticles production in a new Marinomonas strain isolated from the Antarctic psychrophilic ciliate Euplotes focardii
Source: Sci Rep. 2020 Jun 23;10:10218. doi: 10.1038/s41598-020-66878-x (PMC7311414; doi:10.1038/s41598-020-66878-x)
Supplement: Supplementary file 1 — Supplementary information [file 41598_2020_66878_MOESM1_ESM.pdf]

Supplementary materials

Scientific Reports

Horizontal gene transfer and silver nanoparticles production in a new Marinomonas strain isolated from the Antarctic psychrophilic ciliate *Euplotes focardii.*

Maria Sindhura John[1°], Joseph Amruthraj Nagoth[1°], Kesava Priyan Ramasamy[1°], Patrizia Ballarini[1], Matteo Mozzicafreddo[1], Alessio Mancini[1], Andrea Telatin[2], Pietro Liò[3], Gabriele Giuli[4], Antonino Natalello[5], Cristina Miceli[1], Sandra Pucciarelli[1#]

[1] School of Bioscience and Veterinary Medicine, University of Camerino, Via Gentile III da Varano, 1, 62032, Camerino, Italy.

[2] Quadram Institute Bioscience, Gut Microbes and Health Institute Strategic Program, Norwich Research Park, Norwich, UK

[3] Computer Laboratory, University of Cambridge, 15 JJ Thomson Avenue, Cambridge, UK

[4] School of Science and Technology, University of Camerino, Via Gentile III da Varano, 1, 62032, Camerino, Italy.

[5] Department of Biotechnology and Biosciences, University of Milano-Bicocca, Piazza della Scienza, 2, 20126, Milano, Italy.

[#] corresponding author

e-mail: sandra.pucciarelli@unicam.it; tel +39 0737 403231

° equal contribution

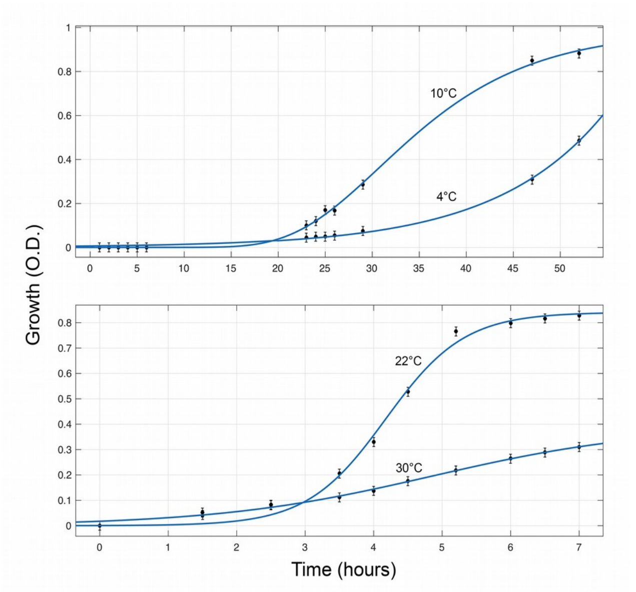

Fig. S1 - Growth curves of *Marinomonas* sp. ef1 in LB medium at 4°C, 10°C, 22°C, and 30 °C. Data represent mean ± standard error of three replicates.

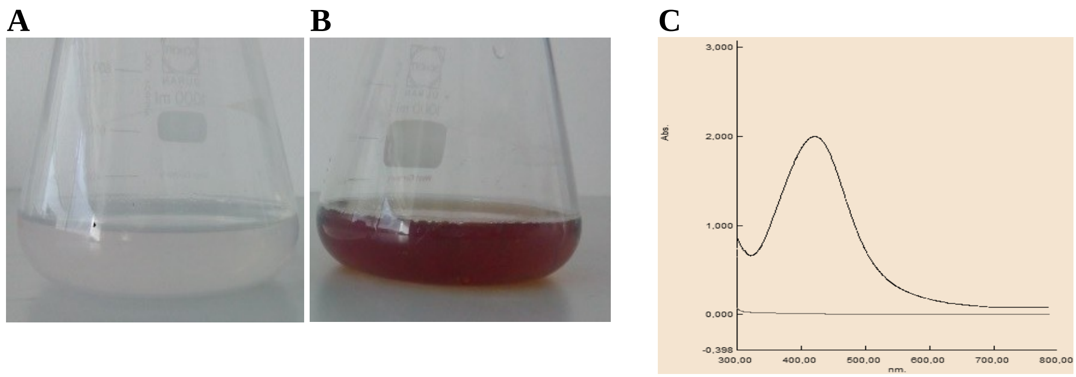

Figure S2: AgNP synthesis by *Marinomonas* sp ef1. A and B: Visual observation of the AgNP synthesis. Medium colour change by the cultured *Marinomonas sp* ef1 from white (A) to dark brown (B) during incubation (24 hrs.) with 1mM of $AgNO_3$. (C) UV–vis spectrum of *Marinomonas* sp ef1 AgNPs. A small aliquot (0.1 ml) of the culture was diluted with ddH2O and UV–visible spectra was recorded from 300 to 800 nm wavelength at room temperature. The peak corresponds to 430 nm.

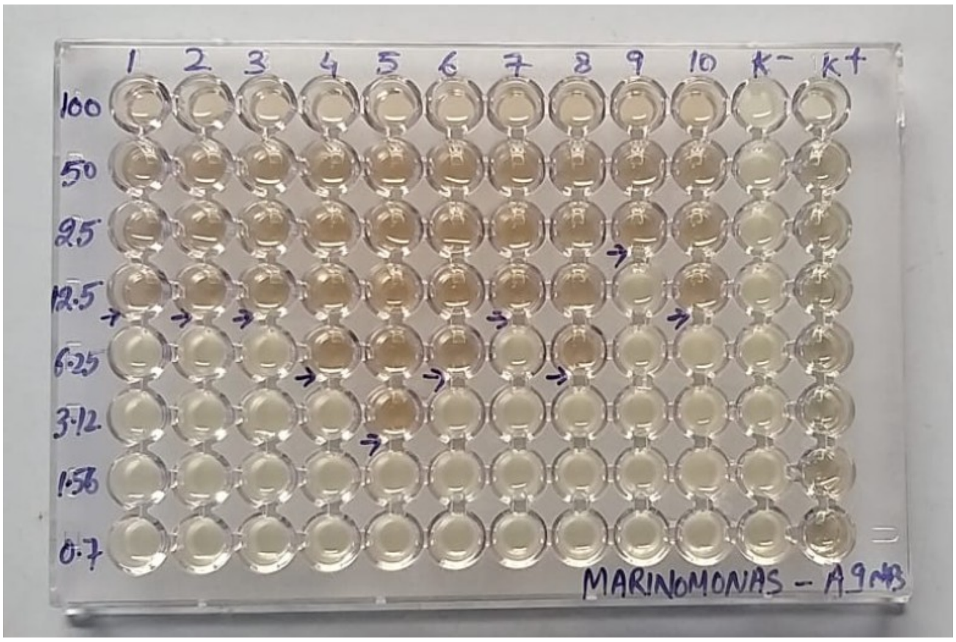

MIC and MBC of AgNps against various pathogenic bacteria

Pathogenic bacterial isolate	Reference isolate	MIC µg/ml	MBC µg/ml
Staphylococcus aureus	ATCC®25923	12.5±0.3	25±0.2
Escherichia coli	ATCC®25922	12.5±0.2	25±0.1
Klebsiella pneumoniae	ATCC®13883	12.5±0.3	25±0.3
Pseudomonas aeruginosa	ATCC®27853	6.25±0.1	12.5±0.2
Proteus mirabilis	ATCC®35659	3.12±0.1	6.25±0.1
Citrobacter koseri	ATCC®25408	6.25±0.2	6.25±0.1
Acinetobacter baumanii	ATCC®19606	12.5±0.2	12.5±0.3
Serratia marcescens	ATCC®13880	6.25±0.3	12.5±0.2
Candida albicans	ATCC®90028	25±0.5	25±0.2
Candida parapsilosis	ATCC®22019	12.5±0.3	25±0.3

Fig S3. Minimum Inhibitory Concentration (MIC) and Minimum Bactericidal Concentration (MBC) assay of *Marinomonas* sp ef1 AgNPs by Broth microdilution method. *1. Staphylococcus aureus 2. Escherichia coli 3. Klebsiella pneumoniae 4. Pseudomonas sp 5. Proteus mirabilis 6. Citrobacter koseri 7. Acinetobacter baumanii 8. Serratia marcescens 9. Candida albicans 10. Candida parapsilosis*

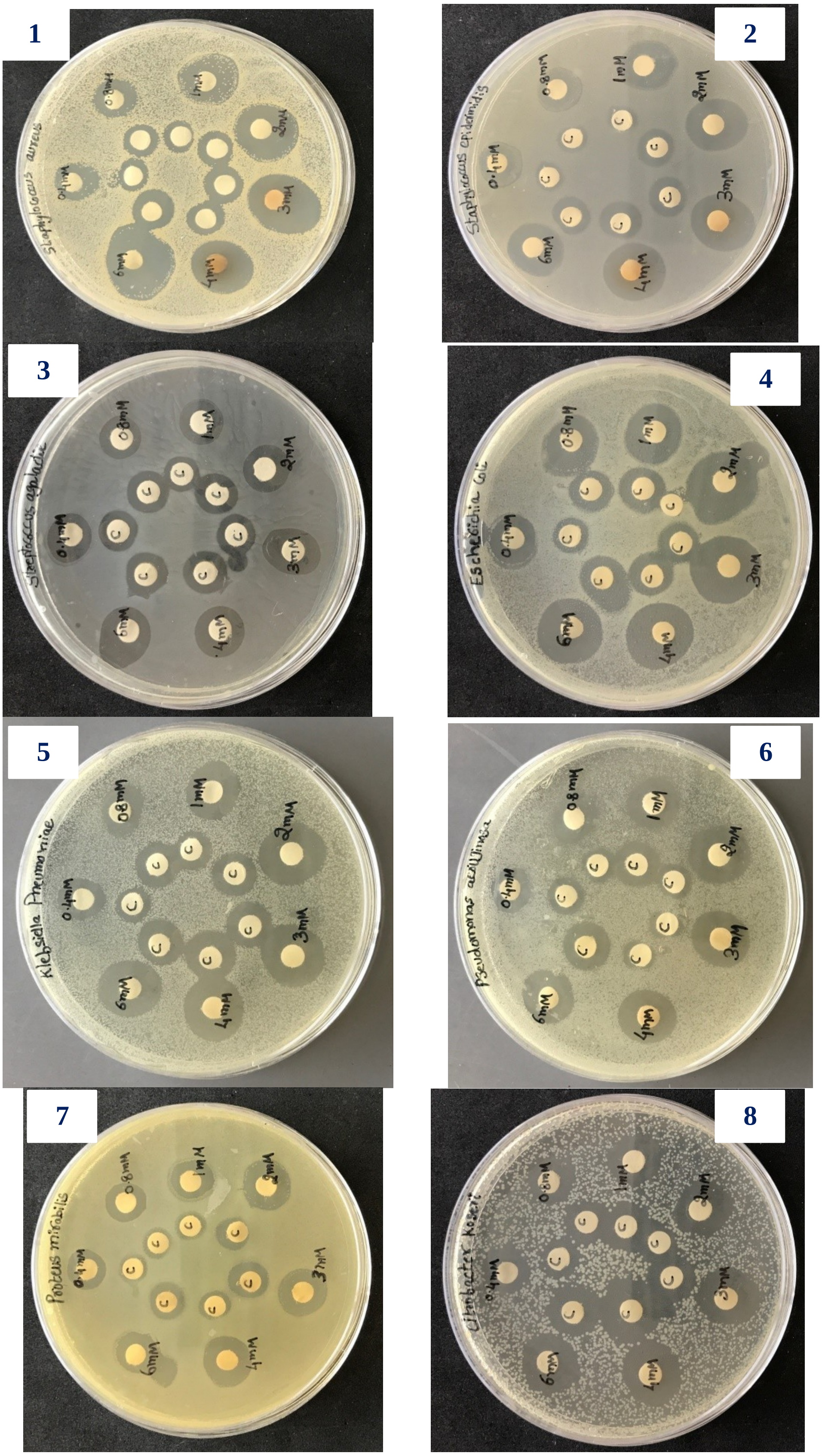
1
2
3
4
5
6
7
8

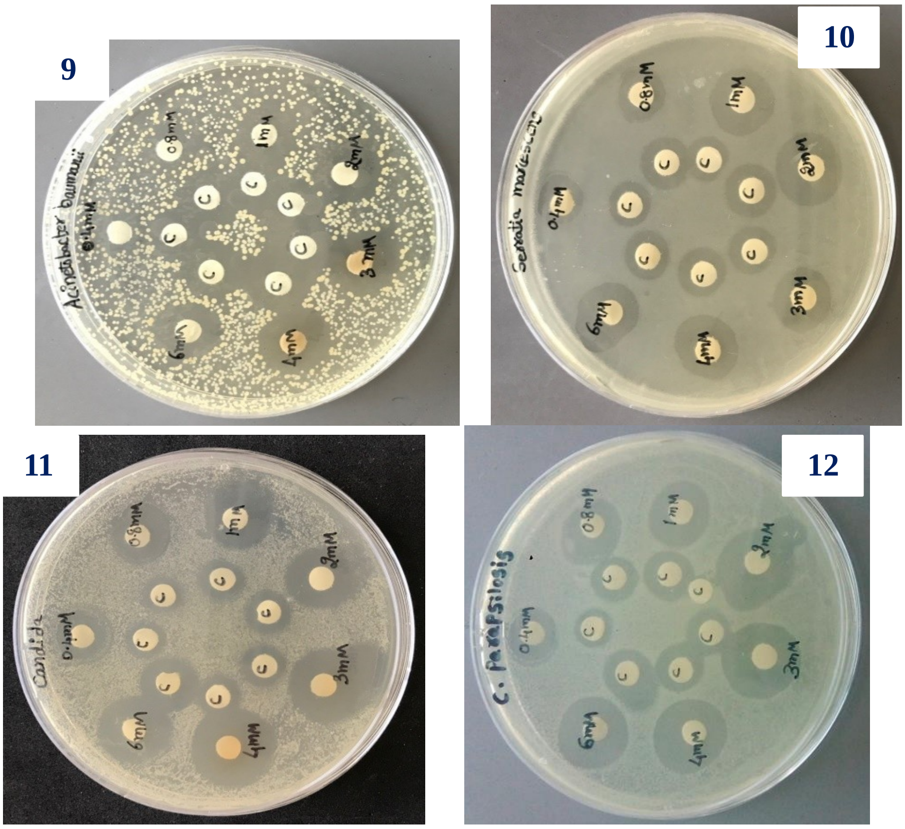

Fig. S3: Antimicrobial activity of increasing concentration (0.4mM, 0.8mM, 1mM, 2mM, 3mM, 4mM and 6mM) of AgNPs tested against twelve human pathogens and compared with increasing concentration (0.4mM, 0.8mM, 1mM, 2mM, 3mM, 4mM and 6mM) of AgNO3. The human pathogens are: *Staphylococcus aureus*, 2. *Staphylococcus epidermidis*, 3. S*treptococcus agalactie*, 4. *Escherichia coli*, 5. *Klebsiella pneumonia*, 6. *Pseudomonas aeruginosa*, 7. *Proteus mirabilis*, 8. *Citrobacter koseri*, 9. *Acinetobacter baumanii*, 10. *Serratia marcescens*, 11. *Candida albicans*, 12. *Candida parapsilosis*.

Supplementary tables

Table S1: List of the transposase genes present in *Marinomonas polaris* and *Marinomonas* sp. ef1

Marinomonas polaris transposases

Sequence Name		Sequence Description
EJ71DRAFT_00421		IS110 family transposase
EJ71DRAFT_01666		IS110 family transposase
EJ71DRAFT_02125		YKGN_ECOLIPUTATIVE PSEUDOGENE: ame: Full= transposase
EJ71DRAFT_02804	-	Streptomyces griseus
EJ71DRAFT_02931		IS110 family transposase
EJ71DRAFT_03199		INH13_ECOLI ame: Full=Transposase for insertion sequence element IS5-13
EJ71DRAFT_03200		INSH5_ECOLI ame: Full=Transposase for insertion sequence element IS5Y
EJ71DRAFT_03752		INSH6_ECOLI ame: Full=Transposase for insertion sequence element IS5H
EJ71DRAFT_03753		IS110 family transposase
EJ71DRAFT_03856		INSN_SHIDY ame: Full=Transposase for insertion sequence element IS911
EJ71DRAFT_03944		YKGN_ECOLIPUTATIVE PSEUDOGENE: ame: Full= transposase
EJ71DRAFT_04184		INSN_SHIDY ame: Full=Transposase for insertion sequence element IS911
EJ71DRAFT_04367		INSN_SHIDY ame: Full=Transposase for insertion sequence element IS911
EJ71DRAFT_04466		INSH5_ECOLI ame: Full=Transposase for insertion sequence element IS5Y
EJ71DRAFT_04469		transposase
EJ71DRAFT_04490		INSH5_ECOLI ame: Full=Transposase for insertion sequence element IS5Y
EJ71DRAFT_04507		INSH5_ECOLI ame: Full=Transposase for insertion sequence element IS5Y
EJ71DRAFT_04544		INSN_SHIDY ame: Full=Transposase for insertion sequence element IS911
EJ71DRAFT_04547		ISSfl4 ORF1
EJ71DRAFT_04592		Transposase DDE domain

Marinomonas sp ef1 transposases

Sequence Name	Sequence Description
M_PROKKA_04338	INSH6_ECOLI ame: Full=Transposase for insertion sequence element IS5H
M_PROKKA_04339	INSH5_ECOLI ame: Full=Transposase for insertion sequence element IS5Y

Table S2: list of ORFs predicted from the *Marinomonas* sp. ef1 genomic islands

GI n.	Gene name	Percentage identity with more similar proteins
**1**	Putative prophage CPS-53 integrase	**Marinomonas sp ef1** 100.00 49.63 50.49 51.84 22.93 24.32 26.01<br>Pantoea_agglomerans_WP_033785581.1 49.63 100.00 51.73 50.82 22.28 25.59 23.98<br>Shewanella_sp._Sh95_KPN78873.1 50.49 51.73 100.00 53.86 23.48 24.94 21.83<br>Vibrio_parahaemolyticus_WP_029807421.1 51.84 50.82 53.86 100.00 21.16 26.37 24.49<br>Marinomonas_atlantica_WP_067098003.1 22.93 22.28 23.48 21.16 100.00 24.14 23.62<br>Marinomonas_spartinae_WP_067011558.1 24.32 25.59 24.94 26.37 24.14 100.00 49.75<br>Marinomonas_polaris_WP_072839418.1 26.01 23.98 21.83 24.49 23.62 49.75 100.00
**2**	Bacterial regulatory proteins, tetR family	**Marinomonas sp ef1** 100.00 52.68 51.47 52.68 18.72 22.50 22.50<br>Pseudomonas_amygdali_KPY77198.1 52.68 100.00 59.31 95.77 22.17 21.50 21.50<br>Methylotenera_versatilis_301_ADI29544.1 51.47 59.31 100.00 59.31 22.28 22.50 22.50<br>Pseudomonas_syringae__coryli_KPW95816.1 52.68 95.77 59.31 100.00 21.67 21.00 21.00<br>Marinomonas_gallaica_WP_067219220.1 18.72 22.17 22.28 21.67 100.00 58.88 58.88<br>Marinomonas_sp._MED121_EAQ65290.1 22.50 21.50 22.50 21.00 58.88 100.00 96.80<br>Marinomonas_sp._S3726_KJZ10839.1 22.50 21.50 22.50 21.00 58.88 96.80 100.00
**2**	NAD(P)H nitroreductase YfkO	**Marinomonas sp ef1** 100.00 60.33 58.68 60.33 61.16 61.16<br>Uliginosibacterium_gangwonense_WP_018607979.1 60.33 100.00 78.51 65.29 65.29 66.94<br>Pseudomonas_batumici_WP_040067214.1 58.68 78.51 100.00 67.77 67.77 68.60<br>Pectobacterium_WP_116166044.1 60.33 65.29 67.77 100.00 72.31 73.55<br>Dickeya_sp._NCPPB 61.16 65.29 67.77 72.31 100.00 96.69<br>Dickeya_sp._Secpp 61.16 66.94 68.60 73.55 96.69 100.00
**2**	Drug resistance protein MdtC	**Marinomonas sp ef1** 100.00 65.20 26.82<br>Ralstonia_solanacearum_WP_071093467.1 65.20 100.00 28.29<br>Marinomonas_spartinae_SBS25146.1 26.82 28.29 100.00
**3**	Xylose operon regulatory protein	**Marinomonas sp ef1** 100.00 46.54 45.69 44.65 32.78 31.05 27.65<br>Verrucomicrobium_BvORR034_WP_050027669.1 46.54 100.00 63.69 65.12 35.43 31.05 29.58<br>Sorangium_cellulosum_KYG04766.1 45.69 63.69 100.00 70.61 37.87 32.79 31.94<br>bacterium_Ellin514_EEF60640.1 44.65 65.12 70.61 100.00 35.43 33.33 29.90<br>Marinomonas_mediterranea_MMB-1_ADZ90985.1 32.78 35.43 37.87 35.43 100.00 52.94 49.21<br>Marinomonas_sp._MED121_EAQ63940.1 31.05 31.05 32.79 33.33 52.94 100.00 45.62<br>Marinomonas_atlantica_WP_067094549.1 27.65 29.58 31.94 29.90 49.21 45.62 100.00
**3**	putative malonic semialdehyde reductase RutE	**Marinomonas sp ef1** 100.00 67.35 67.35 55.61 47.96 44.90 45.41<br>Cystobacter_ferrugineus_OJH41342.1 67.35 100.00 96.94 55.10 50.51 44.90 43.88<br>Cystobacter_fuscus_DSM_2262_EPX60408.1 67.35 96.94 100.00 55.61 49.49 43.88 44.39<br>Vogesella_sp._ 55.61 55.10 55.61 100.00 50.00 46.94 56.12<br>Marinomonas_ushuaiensis_WP_051436225.1 47.96 50.51 49.49 50.00 100.00 75.71 39.50<br>Marinomonas_sp._MED121_EAQ64291.1 44.90 44.90 43.88 46.94 75.71 100.00 37.00<br>Marinomonas_sp._S3726_KJZ08849.1 45.41 43.88 44.39 56.12 39.50 37.00 100.00
**4**	2-keto-4-pentenoate hydratase	**Marinomonas sp ef1** 100.00 54.26 53.49 52.33<br>Amphritea_atlantica_SEQ97558.1 54.26 100.00 61.78 58.69<br>Marinobacterium_lutimaris_SEG65920.1 53.49 61.78 100.00 61.78<br>Nitrincola_nitratireducens_EXJ13042.1 52.33 58.69 61.78 100.00
**4**	Benzene 1,2-dioxygenase subunit beta	**Marinomonas sp ef1** 100.00 65.92 67.04 64.80<br>Marinobacterium 65.92 100.00 79.33 70.95<br>Marinobacterium_georgiense_DSM_11526_SEA66237.1 67.04 79.33 100.00 70.95<br>Amphritea_atlantica_SEQ97596.1 64.80 70.95 70.95 100.00
**4**	Benzene 1,2-dioxygenase subunit alpha	**Marinomonas sp ef1** 100.00 81.92 82.37 82.18 24.18 24.82 25.53 25.29<br>Nitrincola_nitratireducens_EXJ13038.1 81.92 100.00 85.71 84.60 23.71 23.42 24.12 23.89<br>Marinobacterium_georgiense 82.37 85.71 100.00 85.94 25.82 24.59 25.29 25.06<br>Amphritea_atlantica_SEQ97617.1 82.18 84.60 85.94 100.00 25.12 24.12 24.82 24.59<br>Marinobacterium_litorale_WP_027852416.1 24.18 23.71 25.82 25.12 100.00 78.30 79.19 79.19
**5**	Sulfoacetaldehyde reductase	**Marinomonas sp ef1** 100.00 73.31 72.91 72.27 59.76 58.17 58.96<br>Aliagarivorans_marinus_WP_026970871.1 73.31 100.00 98.02 68.92 58.50 57.71 54.18<br>Aliagarivorans_taiwanensis_WP_026958872.1 72.91 98.02 100.00 68.53 57.71 56.52 54.58<br>Psychromonas_arctica_WP_037071234.1 72.27 68.92 68.53 100.00 55.78 56.18 57.37<br>Marinomonas_sp._MED121_EAQ66375.1 59.76 58.50 57.71 55.78 100.00 94.19 75.00<br>Marinomonas_sp._SBI8L_KZM42849.1 58.17 57.71 56.52 56.18 94.19 100.00 75.40<br>Marinomonas_posidonica_AEF55070.1 58.96 54.18 54.58 57.37 75.00 75.40 100.00
**5**	Taurine--pyruvate aminotransferase	**Marinomonas sp ef1** 100.00 79.56 76.70 78.02 64.54 64.54 62.78<br>Agarivorans_albus_MKT_106_GAD02535.1 79.56 100.00 78.95 79.34 62.42 62.64 61.76<br>Psychromonas_ingrahamii_37_ABM01925.1 76.70 78.95 100.00 83.74 61.57 62.34 61.57<br>Psychromonas_arctica_WP_028868217.1 78.02 79.34 83.74 100.00 64.32 63.44 62.33<br>Marinomonas_sp._TW1_KZN13315.1 64.54 62.42 61.57 64.32 100.00 93.26 81.96<br>Marinomonas_posidonica_IVIA-Po-181_AEF55071.1 64.54 62.64 62.34 63.44 93.26 100.00 82.61<br>Marinomonas_sp._SBI8L_KZM42848.1 62.78 61.76 61.57 62.33 81.96 82.61 100.00

Table S3: Comparison of *Marinomonas* sp ef1 AgNPs antimicrobial activity with that of chemical synthesized AgNPs and silver nitrate. 25 µl of 1mM of each solution were tested. Double distilled water was used as a negative control. R*= Resistance

S.No	Microorganism	Bio-AgNps (mm)	Chemical AgNps (mm)	$AgNO_3$ (mm)	Dist.Water (mm)
			**Gram positive bacteria**		
1	Staphylococcus aureus	15±0.4	13±0.5	8±0.3	R
2	Staphylococcus epidermidis	13±0.3	11±0.2	9±0.4	R
3	Streptococcus agalactie	14±0.2	12±0.4	8±0.2	R
			**Gram negative bacteria**		
4	Escherichia coli	17±0.3	14±0.3	11±0.2	R
5	Klebsiella pneumoniae	16±0.1	13±0.2	8±0.4	R
6	Pseudomonas sp	15±0.2	12±0.3	9±0.1	R
7	Proteus mirabilis	14±0.3	12±0.2	8±0.2	R
8	Citrobacter koseri	15±0.1	13±0.3	10±0.4	R
9	Acinetobacter baumanii	14±0.4	11±0.2	10±0.3	R
10	Serratia marcescens	14±0.3	11±0.3	9±0.2	R
			**Fungi**		
11	Candida albicans	14±0.4	11±0.4	8±0.2	R
12	Candida parapsilosis	12±0.2	10±0.2	8±0.2	R

Table S4: Antimicrobial activity of ampicillin and anphoteracin B against pathogenic microorganisms. R*= Resistance

S.No	Microorganism	6 mM (mm)	12 mM (mm)	15 mM (mm)	25 mM (mm)
		**Gram positive bacteria**			
**1**	***Staphylococcus aureus***	18±0.2	21±0.3	25±0.2	27±0.3
**2**	***Staphylococcus epidermidis***	22±0.1	24±0.2	26±0.3	29±0.2
**3**	***Streptococcus agalactie***	13±0.2	16±0.4	18±0.2	22±0.2
		**Gram negative bacteria**			
**4**	***Escherichia coli***	10±0.1	14±0.3	16±0.2	20±0.2
**5**	***Klebsiella pneumoniae***	12±0.1	16±0.2	18±0.2	24±0.1
**6**	***Pseudomonas sp***	18±0.2	22±0.3	24±0.1	28±0.1
**7**	***Proteus mirabilis***	14±0.3	16±0.2	19±0.2	24±0.2
**8**	***Citrobacter koseri***	R	R	R	12±0.2
**9**	***Acinetobacter baumanii***	R	R	R	R

**10**	***Serratia marcescens***	15±0.3	17±0.3	19±0.2	26±0.2

Fungi

S.No	Microorganism	2 mM (mm)	4 mM (mm)	6 mM (mm)	10 mM (mm)
**11**	***Candida albicans***	R	R	12±0.2	22±0.1
**12**	***Candida parapsilosis***	R	13±0.2	16±0.1	24±0.1
